# Recurrent Posterior Strokes in Inflammatory Bowel Disease Patients

**DOI:** 10.1155/2015/672460

**Published:** 2015-02-15

**Authors:** Amir Shaban, Brett Hymel, Maria Chavez-Keatts, Jordan J. Karlitz, Sheryl Martin-Schild

**Affiliations:** ^1^Department of Neurology, University of Iowa, Iowa City, USA; ^2^Department of Gastroenterology, Louisiana State University Health Sciences Center New Orleans, New Orleans, USA; ^3^Department of Neurology, Louisiana State University Health Sciences Center New Orleans, New Orleans, USA; ^4^Department of Internal Medicine, Section of Gastroenterology, Tulane University School of Medicine, New Orleans, USA; ^5^Department of Neurology, Tulane University School of Medicine, New Orleans, USA

## Abstract

*Objective*. To describe the stroke characteristics of patients with a history of inflammatory bowel disease (IBD). *Background*. A hypercoagulable state associated with IBD has been frequently implicated as a risk factor for ischemic stroke. Variable mechanisms and infrequent occurrence limit prospective clinical research on the association between IBD and stroke. *Methods*. We retrospectively reviewed consecutive patients with acute ischemic stroke presenting to our medical center from 7/2008 to 9/2013. Patients with a history of IBD were identified. Clinical variables were abstracted from our prospective stroke registry. *Results*. Over the period of five years we identified only three patients with a documented history of IBD. Each of these patients presented three times to our hospital with new strokes. Patients presented outside the window for intravenous tPA treatment on 8/9 admissions. Each one of our patients had posterior strokes on at least two separate occasions. Hypercoagulation panel showed elevated factor VIII with or without concomitant elevation of Von Willebrand factor (vWF) during almost every admission (8/9 admissions). Only one admission was associated with IBD flare. *Conclusion*. The association between IBD and posterior strokes is a novel finding. Factor VIII elevation may serve as a biomarker of a peristroke hypercoagulable state in patients with IBD.

## 1. Introduction

Inflammatory bowel disease (IBD) comprises two distinct subtypes, ulcerative colitis (UC) and Crohn's disease (CD). These entities are chronic, relapsing, and remitting inflammatory conditions affecting the digestive system. IBD is associated with a hypercoagulable state and an increased risk for thromboembolism, which represent a significant cause of morbidity and mortality in IBD patients [1–5]. Previous studies have shown that platelet abnormalities play an important role in generating the hypercoagulable state [5–8]; it has also been shown that IBD are associated with changes in plasma levels of different hemostatic biomarkers that are consistent with subclinical activation of the coagulation system [4, 9].

Ischemic stroke in patients with IBD occurs through several mechanisms, including large artery disease, small vessel disease, paradoxical embolism, vasculitis, and hypercoagulable state [10, 11]. A systematic review of 5 studies has shown a modest increased risk of stroke among patients with IBD (OR, 1.18; 95% CI, 1.09–1.27) [12]. Other studies suggested that this increased risk of stroke among IBD patients is specific for younger patients <50 years old [12, 13].

Even though many studies have been done to analyze the association between IBD and ischemic strokes, none has studied the stroke characteristics, predicting factors, or outcomes that IBD patients with strokes may have. There are currently no specific recommendations on how to manage these patients and they are usually managed similarly to other stroke patients. It is not clear how IBD-specific treatment impacts the risk of thrombotic events. Variable mechanisms and infrequent occurrence limit prospective clinical research on this disease.

In this case series we describe the clinical characteristics of three IBD patients with nine associated hospital admissions for ischemic stroke.

## 2. Methods

We retrospectively reviewed consecutive patients with acute ischemic strokes presenting to our medical center from 7/2008 to 9/2013. Patients with history of IBD were identified from our prospectively acquired stroke registry and the preexisting diagnosis of IBD was confirmed by chart review [14]. Serial neurologic evaluations including National Institute of Health Stroke Scale (NIHSS) were performed by a trained member of the Stroke Program. Following the initial assessment, patients underwent computed tomography scan and other imaging studies, including echocardiography, as appropriate. Laboratory tests for hypercoagulability were ordered upon admission at the discretion of the treating physician, typically when no obvious cause of stroke was identified through cardiac and vascular imaging and telemetry. Posterior circulation strokes are defined as strokes in the vertebrobasilar circulation territory, including the posterior cerebral artery (PCA) territory, provided a fetal PCA has been excluded. Trained research assistants collected data via chart review. This study was approved by the institutional review board at the Tulane University (IRB protocol number (544540)).

## 3. Results

### 3.1. Case 1

M. S. is a black female with a past history of ulcerative colitis (UC) with two to three flares per year. She was initially evaluated at the age of 42 years for prior cerebellar infarction; at that time she was taking clopidogrel for stroke prevention and prednisone for an ulcerative colitis flare. Three months later, she had a basilar artery occlusion treated with intravenous tissue plasminogen activator (tPA) at an outside hospital with excellent recovery (NIHSS = 0). Anticoagulation was initiated with warfarin.

#### 3.1.1. First Admission

She presented to our service when she was 44 years old, while taking warfarin (INR of 2.5), with headache, left sided numbness, minor facial paresis, and left arm ataxia (NIHSS = 3) outside the 4.5-hour window for treatment with intravenous tPA; however, she received intra-arterial tPA. Angiography showed distal occlusion of left vertebral artery, but no other lines of evidence of vasculopathy or vasculitis. MRI was done and showed bilateral cerebellar acute ischemic strokes. Transesophageal echocardiography did not reveal an embolic source. Her previous strokes were temporally associated with flares of her ulcerative colitis, but she had no active GI symptoms. The treating physician ordered a hypercoagulation panel which revealed a marked elevation in Factor VIII 407% (reference values 50–150%); vWF was not ordered at this presentation. She was found to have a single mutation (C677T) in the MTHFR gene, but her homocysteine was normal. Interestingly, ESR and CRP were not elevated, reducing the likelihood of systemic inflammation of a concomitant UC flare. She was discharged to in-patient rehabilitation on aspirin 325 mg and dabigatran, with NIHSS = 3. Later, the aspirin was discontinued.

Interestingly, three weeks after her stroke, Factor VIII levels decreased from 407% to 215%. Two months later a third measurement was performed; by that time Factor VIII levels were back to normal (133%).

#### 3.1.2. Second Admission

Eight months later, while taking dabigatran and mesalamine for stroke prevention and UC maintenance, the patient presented with left sided arm and leg weakness (NIHSS = 4) again outside the 4.5-hour window for treatment with tPA. MRI was done and showed right cerebellar acute ischemic stroke. Repeat catheter angiography demonstrated a new right superior cerebellar artery occlusion, but no evidence of vasculitis. A hypercoagulation panel was ordered and revealed an elevation in Factor VIII associated with an elevation in vWF. As with her previous presentation, ESR and CRP were not elevated at this presentation. She was discharged home with NIHSS = 4 on dabigatran with additional aspirin.

#### 3.1.3. Third Admission

Two months later, while taking aspirin and dabigatran for stroke prevention and infliximab for UC, the patient presented with left sided weakness (NIHSS = 5) outside the 4.5-hour window for treatment with tPA. An MRI revealed infarction of the right midbrain in the distribution of a paramedian perforator. A hypercoagulation panel was ordered again and revealed an elevation in Factor VIII; vWF level was not ordered at this time. Similar to the previous presentations, ESR and CRP were not elevated at this presentation. She was discharged home with NIHSS = 3 on aspirin, dabigatran, and infliximab.

#### 3.1.4. Subsequent Clinic Visits

She visited our stroke clinic 11 months and then 23 months later for follow-up. She had no neurological symptoms and her UC was quiescent during this period. Her Factor VIII was just above the upper limit of normal at 164.3%.

### 3.2. Case 2

#### 3.2.1. First Admission

E. G. is a fifty-year-old, black female with a past history of Crohn's disease, diagnosed at the age of 19 with subsequent intestinal resection at the age of 21, and a history of ischemic stroke who presented with left sided weakness, mild aphasia, and mild dysarthria (NIHSS = 3) outside the 4.5-hour window for treatment with tPA. At that time she was taking aspirin for stroke prevention and prednisone for a Crohn's disease flare (last flare occurred one month ago). MRI was done and showed right cerebellar and bilateral occipital acute ischemic strokes. MRA revealed a hypoplastic left vertebral artery, but no other vascular abnormalities. Transesophageal echocardiography did not reveal an embolic source. Hypercoagulation panel showed high normal levels of Factor VIII and vWF, but all other tests were normal. ESR and CRP were not elevated which makes it unlikely to have a concomitant Crohn's flare. She was discharged home with NIHSS = 1 on aspirin and clopidogrel for stroke prevention and prednisone for her Crohn's disease.

#### 3.2.2. Second Admission

Ten days after discharge, the patient presented with partial visual field cut, nasolabial flattening, right leg ataxia, and decreased sensation on the left leg and arm (NIHSS = 4). She was not treated with tPA because of her recent stroke. MRI was done and showed additional areas of right occipital acute ischemia. MRA of the brain remained unremarkable. Hypercoagulation panel was ordered and revealed an elevation in Factor VIII; vWF level was not measured at this time. ESR level was normal; however, CRP levels were elevated which may, in addition to a recent episode of loose stool, suggest a flare of her Crohn's disease. She reported episodes of palpitations and lightheadedness, concerning paroxysmal atrial fibrillation; therefore, she was placed on warfarin. She was discharged home with NIHSS = 1.

#### 3.2.3. Third Admission

Two months later, the patient presented again with slurred speech (NIHSS = 5) outside the 4.5-hour window for treatment with tPA. Her INR was subtherapeutic 1.3 on admission. MRI was done and showed right frontal and right caudate acute ischemic stroke. Transesophageal echocardiography did not reveal an embolic source. Hypercoagulation panel was ordered and revealed an elevation in Factor VIII; vWF was not ordered at this time. ESR and CRP were not elevated at this presentation. She was discharged home with NIHSS = 3 on warfarin for stroke prevention and prednisone for her Crohn's disease.

#### 3.2.4. Subsequent Clinic Visits

She visited our stroke clinic 2 and then 8 months later for follow-up. She had no new neurological symptoms or Crohn's disease flares during this period. She continued to receive warfarin and prednisone for stroke and Crohn's disease prevention, respectively.

### 3.3. Case 3

#### 3.3.1. First Admission

J. F. is a sixty-seven-year-old, black female with a past history of Crohn's disease who presented with headache and visual field cut (NIHSS = 5). Her last colonoscopy revealed ulcerations in the transverse colon, descending colon, sigmoid and cecum. She also had a history of multiple ischemic strokes. She was outside of the 4.5-hour window for treatment with tPA when she arrived to our emergency department. CT and MRI showed right PCA territory subacute ischemic infarct. Catheter angiogram was performed and found complete occlusion of the right PCA, but no evidence of vasculopathy or vasculitis. Transesophageal echocardiography revealed no embolic source. Hypercoagulation panel was ordered which revealed a marked elevation in Factor VIII and vWF levels. She had a mildly elevated homocysteine (17.8 *μ*mol/L), but normal MTHFR genes; the rest of the panel was normal. ESR was elevated with the patient reporting abdominal pain and diarrhea consistent with prior flares of her Crohn's disease; however CRP was within normal range. She was discharged home with PT and OT. Warfarin was recommended but the patient refused. She was discharged on clopidogrel and prednisone for her Crohn's disease. Her NIHSS at discharge was 3.

#### 3.3.2. Second Admission

Two months later, the patient presented again with altered mental status and right sided weakness (NIHSS = 29) outside the 4.5-hour window for treatment with tPA. MRI was done and showed left frontal and right occipital acute ischemic strokes. Hypercoagulation panel was ordered again and revealed an elevation in Factor VIII; vWF was not measured at this time. ESR and CRP were not ordered at this presentation. The patient refused warfarin and was discharged home on clopidogrel with NIHSS = 3. More intensive treatment for her Crohn's disease was recommended, but she was tolerant of her GI symptoms and resistant to further evaluation and treatment.

#### 3.3.3. Third Admission

Five months later, the patient presented again with slurred speech (NIHSS = 9) outside the 4.5-hour window for treatment with tPA. MRI was done and showed left MCA acute ischemic stroke. A hypercoagulation panel was ordered again and revealed an elevation in Factor VIII; vWF was not ordered at this time. Similar to the previous presentations, ESR and CRP were not elevated. The patient agreed to take warfarin and she was discharged home with PT and OT. Her NIHSS at discharge equals 6.

Interestingly, Factor VIII levels dropped from 400% on her first admission to 200% when measured a year later in the clinic setting.

#### 3.3.4. Subsequent Clinic Visits

She visited our stroke clinic 5 months later for follow-up. She had no new neurological symptoms or Crohn's disease flares during this period. She continued to receive warfarin.

All patients had what constitutes our complete hypercoagulable panel during at least one admission for acute stroke. This panel includes full anti-phospholipid antibodies (lupus anti-coagulant, B2 glycoprotein, anti-cardiolipins, and anti-phosphatidylserine antibodies), vWF, homocysteine (as well as MTHFR gene, if elevated), lipoprotein A, antithrombin III, protein C, protein S, Factor V Leiden screen, prothrombin gene, ANA, and ANA profile. Only abnormal lab tests were repeated during subsequent admissions.

## 4. Discussion

Of 1382 patients admitted 3 patients (0.2%) had a history of IBD. Our study is the first to present three patients with history of IBD suffering from recurrent strokes.

Each of our patients had at least three ischemic strokes within a one year period. There have been no previous studies investigating the association between a history of IBD and recurrent strokes. However, it has been established that IBD is associated with a hypercoagulable state which in turn may cause recurrent strokes [14]. For this reason, secondary stroke prevention may be of much greater benefit for patients with history of IBD, once they had one; these patients are expected to have subsequent strokes. The optimal medication for secondary stroke prevention for IBD patients is yet to be determined but may require optimization of IBD and anticoagulation.

Interestingly each one of our patients had a posterior distribution of stroke on at least two separate occasions. None of our patients had fetal PCA, but two of the three had intact posterior communicating arteries. The frequency of posterior circulation strokes in our entire cohort was 28.2%. This association between IBD and posterior strokes has never been demonstrated; moreover, there is currently no data available in the literature that a hypercoagulable state in general may cause such a predilection for posterior circulation strokes. This phenomenon could be explained by slower blood flow in the posterior circulation which may decrease washing out the activated procoagulation factors and increase the risk of clotting in patients with hypercoagulable state compared to the faster blood flow in the anterior circulation [15, 16].

Knowledge of stroke location is crucial for stroke prevention. Posterior ischemic strokes are known to have a more insidious presentation than anterior strokes. This insidious presentation may result in delay in seeking medical attention decreasing tPA treatment rates for these patients. None of the patients received tPA treatment as a result of presenting outside the window on 8/9 admissions; one presentation was inside the window but tPA was contraindicated due to previous stroke within 3 months. Stroke education with focused posterior stroke symptoms review may be of a great benefit to bring these patients with IBD-associated stroke in time for tPA treatment.

There is conflicting data about the role of IBD as a risk factor for stroke. While some studies have demonstrated that IBD only increases the risk of stroke in young patients, other studies successfully showed an increased risk of stroke associated with IBD in general. Several studies have demonstrated that active disease is associated with increased risk of stroke [10, 11, 17]. Acute ischemic stroke episodes in our study were often not associated with IBD flares.

Factor VIII was elevated on 8/9 admissions; after the stroke Factor VIII levels tended to gradually decrease, suggesting that Factor VIII may be a potential marker of increased risk of stroke in IBD patients. Previous studies showed that elevated FVIII is a risk factor for acute ischemic stroke [18, 19]. Further studies are needed to validate and investigate the sensitivity and specificity of Factor VIII in predicting new strokes in IBD patients.

Our study is limited by the sample size and its retrospective nature which restrict our ability to conduct a valid statistical analysis. Also due to the retrospective nature of this study no investigation for flaring up of the IBD was done apart from CRP, ESR, and clinical examination. Our patients were provided with clinic follow-up appointments with our gastroenterologists. We only describe stroke characteristics in the patients involved in this study and acknowledge that these results may be acquired by chance. However, IBD related strokes have been poorly studied; only case reports are available in the literature. This is likely due to the low prevalence of IBD (in the order of 1/10^5^) and the low rate of stroke in IBD patients (approximately 2-3% per year). Our study results may prompt larger studies. Further studies should investigate the association between hypercoagulable state with posterior circulation strokes and the validity of Factor VIII levels in predicting strokes in patients with history of IBD.

## 5. Conclusions

Inflammatory bowel disease is associated with a poorly defined hypercoagulable state and thrombosis. Factor VIII, an inflammatory marker and prothrombotic protein, was frequently detected at elevated levels in our patients with IBD and stroke. These patients with IBD experienced posterior circulation strokes out of proportion to what is seen in our sample of stroke patients. Further investigation of the role of Factor VIII levels in patients with IBD and posterior circulation susceptibility is necessary.
